# Editorial: Targeting Lipid Rafts as a Strategy Against Infection and Cancer

**DOI:** 10.3389/fcell.2021.748905

**Published:** 2021-09-06

**Authors:** Tina Garofalo, Roberta Misasi, Giulio Preta

**Affiliations:** ^1^Department of Experimental Medicine, “Sapienza” University, Rome, Italy; ^2^Institute of Biochemistry, Life Science Center, Vilnius University, Vilnius, Lithuania

**Keywords:** lipid rafts, cholesterol, cancer therapy, membrane lipid bilayer, infection

The concept of specialized “membrane microdomains” (MM), often referred as lipid rafts, has extensively influenced the molecular biology of plasma membrane over the last decades. These cholesterol/sphingolipid-rich domains, play an essential role in the regulation of cellular processes including intracellular signaling, cell death, and redox homeostasis (Simons and Toomre, 2000; Mollinedo and Gajate, 2015). The involvement of MM in the pathogenesis of several conditions has been elucidated over the last years, leading to the development of innovative pharmacological approaches, specifically targeting their components, including both lipids and proteins. The specific interactions between the various classes of molecules give lipid rafts some peculiar properties, both physical and biochemical. Indeed, the physicochemical basis of raft hypothesis was derived by several studies on model membranes, where mixture of lipids, resembling the composition of the outer plasma membrane, segregates in liquid ordered and disordered domains with distinct characteristics (Brown and London, 1998; Simons and Vaz, 2004). The effects of different drugs on membrane properties were unveiled using artificial membranes, putting the basis for new therapeutic strategies based on modification of membrane biophysical properties (Peetla et al., 2009; Knobloch et al., 2015). Statins are the ideal example of how this innovative approach connects with the classical strategy based on membrane cholesterol depletion. Statins, a well-known class of cholesterol lowering agents, possess several pleiotropic effects (i.e., cholesterol-independent), including the ability to influence the organization of artificial and biological membranes (Wang et al., 2008; Redondo-Morata et al., 2016; Galiullina et al., 2019; Penkauskas et al., 2020). For this reason, they are increasingly used to enhance delivery and efficiency of chemotherapeutic drugs (Pinzon-Daza et al., 2012; Di Bello et al., 2020). In his minireview, Preta summarizes cancer therapeutic strategies based on altering membrane cholesterol/sphingolipid content as well on changing cancer membrane bilayer properties, as fluidity or thickness, with the final aim to increase sensitivity to cytotoxic drugs and defeat multidrug resistance. Vona et al. review provides an update on cholesterol-targeting strategies based on inhibition of its synthesis, modulation of its uptake and intracellular transport and on the possibility of therapeutic intervention in the treatment and/or prevention of certain types of cancer.

However, the exclusive properties of lipid rafts and their vital importance for the dynamics of a cell, make them susceptible to pathogens hijacking. Indeed, many steps of pathogen interaction with host cells rely on host lipid rafts and in several cases this interaction lead to microdomains modifications. During bacterial infections, many toxins interact with membrane rafts. Yeh et al. report the ability of *Campylobacter jejuni* cytolethal distending toxin (CDT) in reducing the effect of two other lipid rafts-binding cytotoxins, vacuolating cytotoxin A (VacA), and cytotoxin-associated gene A (CagA) involved in *H. pylori* disease progression. The authors demonstrate that CDT is capable to hijack cholesterol, competing with the other two toxins and drastically mitigating *H. pylori* pathogenesis. Mergani et al. outline the modifications of lipid rafts-dependent sorting of sucrose isomaltase during *Staphylococcus aureus* infection, elucidating the molecular mechanisms associated with the bacterial alterations of intestinal functions. Sucrose isomaltase is the major intestinal α-glucosidase responsible for catalyzing the hydrolysis of dietary carbohydrates and its deficiency/malfunction is often associated with gastro-intestinal symptoms.

In their review Sorice et al. describe how lipid rafts provides a multimolecular platform to segregate the angiotensin-converting enzyme (ACE-2) receptor, the main receptor of SARS-CoV-2. If we take into consideration the probable hypothesis that the fundamental contact for the entry of the virus into the host cell occurs at the level of these MM, lipid rafts-targeting drugs, alone or in combination with other compounds may play an antiviral role (Fecchi et al., 2020). Statins (by inhibition of cholesterol synthesis) or cyclodextrins (by depletion of membrane cholesterol), affecting cholesterol levels and disrupting lipid rafts, could effectively inhibit coronavirus adhesion and binding, preventing the progression of the virus (Baglivo et al., 2020). Indeed, according to recent observational studies, statins were effective in reducing the severity or mortality of COVID-19 (Kow and Hasan, 2020; Zhang et al., 2020). In the same way, cyclodextrins have been extensively used to increase stability, solubility and bioavailability of many drugs. From this point of view, one of the most interesting hypothesis is the one formulated in a recent letter which reports the mechanism of cyclodextrin- soluble angiotensin - converting enzyme 2 (CD-sACE2) inclusion compounds in the treatment of SARS-CoV-2 infections (Sun et al., 2020).

In conclusion the progress made in the last years in understanding the biological significance of lipid rafts is indisputable, however few gray areas remain to be further investigated including the interplay between lipids and membrane proteins in regulating membrane organization and how targeting these rafts proteins could provide new therapeutic strategies. Continuous advancement in microscopic techniques could largely contribute to this emerging investigational field and potentially allow a direct observation of membrane domains interactions *in vivo* (Levental et al., 2020). We hope that all the information reported in this Research Topic will be useful to researchers in this exciting field, and will push further studies aimed to test new effective membrane-lipid targeting agents. We want to acknowledge the great work of the authors, co-authors, and reviewers, and to thanks the support constantly received from Frontiers Team members.

## Author Contributions

TG, RM, and GP wrote and corrected the manuscript. All authors contributed to the article and approved the submitted version.

## Conflict of Interest

The authors declare that the research was conducted in the absence of any commercial or financial relationships that could be construed as a potential conflict of interest.

## Publisher's Note

All claims expressed in this article are solely those of the authors and do not necessarily represent those of their affiliated organizations, or those of the publisher, the editors and the reviewers. Any product that may be evaluated in this article, or claim that may be made by its manufacturer, is not guaranteed or endorsed by the publisher.
